# A Bilingual Advantage? An Appeal for a Change in Perspective and Recommendations for Future Research

**DOI:** 10.3390/bs9090095

**Published:** 2019-09-04

**Authors:** Gregory J. Poarch, Andrea Krott

**Affiliations:** 1 English Department, University of Münster, 48143 Münster, Germany; 2 School of Psychology, University of Birmingham, Birmingham B15 2TT, UK

**Keywords:** executive function, cognitive effects, bilingual advantage, modulating factors

## Abstract

The debate on possible cognitive advantages bilinguals have over monolinguals continues to occupy the research community. There is an ever-growing research body focusing on adjudicating whether there is, in fact, an effect of using two or more languages regularly on cognition. In this paper, we briefly review some of the more pertinent literature that has attempted to identify attenuating, modulating, and confounding factors in research comparing monolingual and bilingual populations, and we highlight issues that should be taken into account in future research to move forward as a research community. At the same time, we argue for a change in perspective concerning what is deemed an advantage and what is not and argue for more ecologically valid research that investigates real-life advantages.

## 1. Introduction

The notion of a bilingual advantage on cognition, driven by a lifelong usage of multiple languages, has become an increasingly debated topic. From the beginning of the 1990s, the number of studies comparing monolingual and bilingual and/or multilingual populations has seen a steady increase [1]. While there is ample research reporting significant differences across groups in favour of better performance of bilinguals (in children: [2,3,4,5]; in young adults: [6,7,8]; in older adults: [9]; see reviews [10,11]), at least in some aspects of cognitive control, there are now also a number of studies that have found no differences across groups [12,13,14,15], or even better performance by monolinguals [14]. This has led to the ongoing debate about whether or not the reported differences between bilinguals and monolinguals in favour of bilinguals, coined the bilingual advantage, are in fact real. In this paper, we address the ongoing debate by giving a brief non-exhaustive overview of recent work in the field, by discussing whether the term ‘bilingual advantage’ is appropriate and for what kind of finding, and by identifying how the research community could move forward and re-frame the research questions at hand.

## 2. The Bilingual Advantage

Early views on bilingualism were that of it being a debilitating factor [16]. These views prevailed until the 1960s research conducted with children in Canada [17], a turn that to some extent may have been language policy-driven [18]. The early detrimental views could also have an influence on the present debate in that there may still be the urge to defend bilingualism as having more positive traits than negative, both from an individual and a socially-relevant perspective.

From these early bilingualism studies arose a research stream in the late 1980s focused on exploring whether using multiple languages in daily life has effects on the cognitive system. The main focus of these studies was on examining selective attention and metalinguistic development in monolingual and bilingual children [19,20,21] and the results, indicating a bilingual edge, were later used to coin the term ‘bilingual advantage’.

The view that bilinguals could profit cognitively from their bilingualism is based on the theoretical assumption that bilingual and multilingual individuals experience constant cross-linguistic activation and interaction during language processing (see, e.g., [22,23]). Hence, in order to be able to use the correct language in any given situation, there is a need for a cognitive control mechanism that allows speakers to resolve the conflict between actively competing languages. For non-verbal processing in humans, such a cognitive control mechanism already exists—the so-called executive function (EF).

EF is the cognitive control system that individuals employ to make choices between alternative and (sometimes) competing responses in their daily lives [24]. There are several cognitive functions subsumed under the term executive function, amongst which are selective attention, information updating, set-shifting, task monitoring, and conflict resolution [25,26,27]. The development of EF is assumed to start in early childhood up until adolescence, during which it reaches maturity [28].

Several factors have been identified as modulating the development of EF, the most prominent of which is socio-economic status (SES; [29,30]). Other relevant factors that pertain to individuals’ lifestyles are physical activity [31], circadian rhythm [32], sleep [33], dietary intake [34], and musical expertise [35,36]. Furthermore, culture has been shown to have differential effects on EF development (see, e.g., [37,38]). Finally, a relevant factor may be the regular use of multiple languages [39], a notion, as indicated above, that has become highly controversial (cf. [40]).

However, there is evidence for a necessity in bilinguals to draw on EF during language processing [41] or when switching between languages [42]. Such repetitive cognitive control training may over time have an impact on its efficacy [43] and on the neural networks responsible for EF [44] (see also reviews [11,45]).

## 3. How is Non-Verbal Cognitive Processing Measured in Experimental Research?

There are a number of experimental paradigms that tap non-verbal cognitive processes. For present purposes, we limit ourselves to three tasks that have been used ubiquitously in the field of research on multilingualism and EF—the Flanker task [46], the Simon task [47], and the Colour–Shape switching task [48].

The Flanker and the Simon task are thought to induce cognitive conflict during task performance, requiring selective attention to identify conflict and subsequent cognitive resources for conflict resolution, albeit in slightly different manners. While the Flanker task uses arrays of arrows that are either congruent or incongruent to measure resistance to the interference of flanking distractors [49], the Simon task uses coloured squares to induce conflict by a spatial–stimulus-response mismatch in incongruent trials compared to an absence of a mismatch in congruent trials. In both tasks, beyond inspecting overall reaction times in the congruent and incongruent conditions, a difference score as an index of inhibitory control is calculated (the congruent condition reaction time subtracted from the incongruent condition reaction time). The difference score magnitude indicates how strongly distracted individuals are in the incongruent condition compared to the congruent condition. A larger magnitude indexes poorer interference control (for a more detailed account of how performance in these tasks can be modelled, see [24,25]).

The question of whether or not bilingual speakers differ from monolingual speakers in terms of task switching has most frequently been tested by means of the Colour–Shape switching task [48]. In this task, participants are typically presented with red and green circles and triangles, one at a time. They are asked to either make a shape (circle versus triangle) or a colour (red versus green) decision, depending on a visual cue that either precedes or co-occurs with a stimulus. Flexibility in task switching is measured by the switch cost, which is the reaction time difference between switch and non-switch trials.

## 4. Selected Research Findings Across the Life Span

In this section, we briefly report selected recent studies with participants across the life span, namely with children, young adults, and older adults. Note that the focus here is to highlight the rationale for this paper much more so than to offer a comprehensive review (for reviews, see [11,45]).

In a longitudinal study conducted with 3-year-olds from three countries (Argentina, Vietnam, and the USA), Tran and colleagues [38] found that culture interacted with bilingualism in modulating performance on the Attention Network Task (ANT), a more elaborate variant of the Flanker task [50], adding culture as yet another factor that can be drawn on to explain mixed findings in the literature. Similarly, Park and colleagues [51] tested 8–12-year-olds longitudinally using the Flanker task and a Colour–Shape switching task (the Dimensional Change Card Sort; [52]). Over the course of one year, the bilingual children showed a steep improvement of inhibition, while the monolingual children’s inhibition remained stable. For task switching, no group differences were found, which contrasts earlier findings [53,54]. Thus, “bilingual experience may modulate the developmental rates of some components of EF but not others, resulting in specific EF performance differences between bilinguals and monolinguals only at certain developmental time points.” [51] (p. 1842). This interpretation resonates with that offered by Poarch [55], who found no differences in Simon task performance between L2 and L3 learners aged 5–13, but clear between-group differences in inhibitory control in the Flanker task. These studies also represent a move in the research field towards capturing the development of EF through longitudinal designs (see also Section 8 below “Recommendation for the research field”)

In research with young adults, Naeem et al. [56] had 18–30-year-olds perform a Simon task and reported inconclusive differences between groups of monolinguals and bilinguals. The authors identified differences in socioeconomic status (SES) as the decisive modulating factor in EF task performance, particularly so in low-status individuals. As such, the authors conclude that their “findings run counter to the central assertion of the bilingual advantage account” [56] (p. 1). In studies using switching tasks, smaller switch costs have been reported for bilinguals compared to monolinguals [48,57,58,59,60], but not always [15,61] (for recent reviews, see [60,62]). Prior and Gollan’s [59] findings suggest that enhanced switching performance only holds for bilinguals who frequently switch languages, but this was not confirmed by [15]. Note, however, that the participants in [15] were not assessed on their daily language switching behaviour.

In contrast to young adults, research with older adults has found the superior performance of bilingual individuals in classic EF tasks more consistently (for reviews, see [63,64]). It has been suggested that continuously speaking two languages might affect language control systems located at prefrontal cortices and therefore protect brain areas that are most vulnerable to aging. Possibly the most impressive are the findings that suggest that bilingualism delays the onset of dementia [65,66], and that it leads to a better cognitive outcome after stroke [67,68]. However, as with the cognitive benefit as such, not all studies have found this specific benefit, especially prospective studies that followed healthy adults in contrast to retrospective studies that investigated the onset of dementia in dementia patients (for recent review, see [69]). In order to bring this area of research forward, studies will need to take into account more detailed information on the individual profile of the bilinguals, especially of their language usage. Furthermore, we need more longitudinal studies that can closely track the relationship between cognitive decline and language usage.

## 5. Neural Differences

As the research on the effect of speaking another language on the onset of dementia and recovery from stroke already suggests, bilingualism has implications for brain structure and function. This should not come as a surprise when considering the evidence for experience-based neuroplasticity in other areas, such as for taxi drivers [70] or musicians [71]. More important for the present question, though, are findings of structural changes that arise due to the learning of an additional language, both in terms of the volume of particular brain areas and brain connectivity. Findings in this area are mixed, but the most recent review of the literature by Pliatsikas [72] proposes that bilingual experiences such as immersion or age of acquisition of the additional language play a strong role in neural restructuring [73]. Models that have tried to capture the variability in the findings have proposed continuous changes over the course of bilingual experience [74] and an increase in reliance on posterior and subcortical regions. Most recently, Pliatsikas [72] suggested a dynamic restructuring model, which links brain restructuring to the quantity and quality of exposure to a bilingual environment. For instance, Pliatsikas notes that during the early exposure to a new language grey matter changes seem to occur in anterior regions related to executive control; these changes are not found during the following consolidation stage, most likely due to pruning to the most efficient connections. Importantly, research on the effect of bilingualism on brain structure suggests that bilingualism should be viewed as a continuous adaptation that depends on individual experience. These adaptations are best studied in longitudinal designs.

Changes in brain structure and function have also been linked to behaviour in EF tasks. For instance, Olsen et al. [75] reported that the frontal lobe white matter volume of bilingual participants was positively correlated with performance in an executive function inhibition task (Stroop task), and Gold and colleagues [76] found a relationship between the recruitment of left lateral frontal cortex and cingulate cortex with better performance in bilingual older participants in a task-switching paradigm. Functional differences have also been found with the means of electroencephalographic (EEG) recordings [74,77,78], but usually not accompanied with behavioural differences between monolingual and bilingual participant groups. For instance, Kousaie and Phillips [78] tested monolingual and bilingual participants on a Simon, Flanker, and Stroop task. While they did not find any behavioural differences between the participant groups, they found differences in the EEG signals, albeit not the same for the three tasks. Grundy et al. [74] found greater signal complexity at occipital areas in bilingual than monolingual participants in a switching paradigm. They also found that only the performance of monolinguals was related to occipital–frontal neural coupling. Their results suggest that the brains of monolinguals and bilinguals work differently when performing a switching task.

## 6. Research Summary

The research briefly reviewed above and previous work on cognitive differences between monolinguals and bilinguals used classical EF tasks such as the Flanker task and the Simon task. Many of these studies yielded a systematic difference between groups, some did not, even in the presence of structural or functional brain differences. One interesting fact is that, while there are many studies that show a bilingual advantage and a growing number of studies that show the equal performance of monolinguals and bilinguals, it is rare that monolinguals are reported to outperform bilinguals (for a recent review, see [79]). If all reports of bilingual advantages were simply false positives, one would expect an equal number of false positives of a monolingual advantage. It, therefore, seems to be the case that the groups of monolinguals and bilinguals overlap in terms of their executive function performance and that we have not yet understood precisely which bilinguals outperform which monolinguals (see [80] for a similar view).

As such, we thus dare to ask the question whether the effects found in numerous studies along the way [4,5,55,81,82,83,84,85] (for reviews, see [64,86,87]) should be necessarily deemed an ‘advantage’ of one group over the other. Alternatively, one could consider such research outcomes as systematic differences between two large groups of populations, even if differences are not always found (see [12,13,14] for null-results). Such differences could arise because of a multitude of individual factors on cognitive control abilities and that these factors restructure the brain and its functionality in different ways during a continuous experience with bilingual environments. It could also be due to a false assumption: As researchers we assume that the populations we test differ on only one variable, namely, that one of the groups uses one language only in their daily lives while the other uses more than one language in their daily lives. However, such an assumption is becoming more and more difficult to maintain as individuals are exposed to more and more foreign language input in the media [88] and most learn a second language at school, even if not all reach a high level of proficiency.

If we accept that there are monolingual and bilingual groups and that they show systematic differences in executive function task performance, a different, possibly even more pertinent question arises, namely whether such differences constitute an advantage in real life. For instance, a 30 ms difference in effect magnitude between bilingual and monolingual children’s performance in the Flanker task [55] yielded a significant advantage in inhibitory control for the bilingual over the monolingual children. However, does this difference constitute a significant advantage in real life? In order to answer this question, we need to consider research that has investigated real-life consequences of bilingualism and studies that go beyond testing executive function per se.

## 7. Cognitive Advantages in Real Life

For one, it has been found that precocious EF development in bilingual children from birth may help to offset SES disadvantages [89,90,91]). Furthermore, an enhanced bilingual performance has not only been found for performance in classical EF tasks such as the Simon, Flanker or Colour–Shape switching task, but also, for instance, in perspective taking [92], creative and divergent thinking [93], open-mindedness and cultural empathy [94], or tolerance of ambiguity [95]. Performance differences in such tasks might be indicative of more important advantages in real life.

Let us take a look at one such area of research in more detail, namely that of cognitive flexibility in perspective-taking in a wider sense. Studies have found a bilingual advantage in the theory of mind and perspective-taking tasks [96,97] (see also meta-analysis by Schroeder [92]). For instance, Goetz [96] found that bilingual children performed better in an appearance reality test, a visual perspective-taking task and two false belief tasks. The appearance reality task tested whether children understand the difference between what an object looks like and what it really is (e.g., a pen that looks like a fish). In the perspective-taking task, children needed to understand that somebody else sees an object in a different way. For instance, a picture placed between them and a second person on a table appeared upright to them, but upside down to the other person. As one of the false belief tasks Goetz used a version of the “Sally Anne task” [98], which tests children’s ability to distinguish between their own knowledge/belief and that of others. More specifically, a child is tested on a scenario where a third person has seen and therefore believes an object at a location A, while the child knows that the object has moved to location B in the third person’s absence.

The advanced performance of bilingual children in perspective-taking tasks has been related to EF [97]. However, the role of EF has been questioned. For instance, recently Diaz and Farrar [99] have argued that bilinguals’ false-belief advantage is due to their advanced metalinguistic awareness instead of EF. Furthermore, Fan and colleagues [100] have presented evidence that the bilingual perspective-taking advantage might be due to advanced socio-pragmatic skills instead of advanced EF. It therefore still needs to be shown in how far the enhanced perspective-taking skill is due to a difference in executive function and/or due to some other difference in cognition [93].

In summary, we in the research field may need to assess the relevance of other systematic cognitive differences that go above and beyond the undoubtedly general benefits of being fluent in multiple languages and that may have a more visible impact on multilinguals’ daily lives.

## 8. Recommendation for the Research Field

Given the somewhat mixed results in the field of bilingualism and EF, there is a need to identify ways in which to move forward. In what follows, we list a number of suggestions that may assist in achieving this goal.

(1) Longitudinal studies

As argued above, more longitudinal studies should be run in which the development of non-verbal cognitive control and verbal skills is traced—both in children [38,51], whose cognitive control continuously develops up until adolescence [28] and in older adults, who show decreasing cognitive control with increasing age [101].

(2) The nature of executive function tasks

As outlined above, past research in the field of bilingualism and EF has relied heavily on comparing groups of bilinguals and monolinguals using the prevalent tasks tapping EF such as the Flanker and the Simon task (see Section “How is non-verbal cognitive processing measured in experimental research?”). There are several reasons why this approach may need to be re-considered. First, their very nature as experimental tasks performed in a lab displays a lack in ecological validity, and, second, they have been found to display inconsistent convergent validity [15,24,55,64,78]. Third, different tasks show differences in how conflict is elicited [102] and do not engage fully-overlapping cognitive processes as shown in different neural reflections of interference control [103]. Finally, task performance induces varying cognitive loads, which may play a particularly relevant role in comparisons of bilinguals vs. monolinguals [104], in young children [105], whose executive function subcomponents, as mentioned above, are still in development [28], and in older adults, whose executive function abilities are waning [106]. A move towards using age-appropriate, real-life tasks tapping clearly delineated EF components may thus be necessary.

(3) The content and procedure of executive function tasks

There is also no indication that the tasks tapping EF are implemented in a standardized fashion across studies. While this may be true for general research on EF, it could be a decisive confound in research exploring (subtle) EF differences between bilingual and monolingual populations. For instance, the Flanker task for young children is sometimes run with drawings of fish instead of arrows as stimuli [107]. The Simon task has no fixed colour, size, and on-screen position for the displayed squares. Cues in the switching tasks are either presented before or together with targets. These factors could have an influence on task performance and may or may not be an added confounding factor along with the array of others that have been brought forward. Additionally, the overall number of trials, as well as the ratio between congruent and incongruent trials, differs across studies in various ways [104]. Furthermore, the manner in which the collected data is trimmed (i.e., how outliers are identified and subsequently excluded from further analysis) can obscure possibly relevant differences between groups [83,108], especially if effects might be driven by a subset of data, for instance, slower responses [109]. Finally, the choice of statistical analyses can influence how performance patterns either differ or not [109,110] and may need to be standardized in order to make studies fully comparable. These differences in experimental set-up, stimulus selection and design, procedure, and data processing and analysis may be adding to the variability in research findings. If we as a research field want to be able to interpret research findings uniformly, then we may need to negotiate a fixed manner in which experimental paradigms are developed, executed, and analysed. All these factors may inevitably lead to researchers choosing to run pre-registered studies, which in turn could counteract the reproducibility crisis evident in psychological science research in general [111].

(4) Move away from group designs

As evident in our brief research overview, the typical study investigating the effect of speaking an additional language compares bilingual with monolingual speakers. Against the backdrop of ever more non-homogeneous participant groups and the increasingly problematic distribution of individuals into dichotomous groups of purely monolinguals and bilinguals/multilinguals, the time may have come to disregard group designs. This is all the more relevant given that with increasing age, individuals have ever-growing life and language experiences that may inevitably lead to much greater overlap of groups in terms of background variables than previously assumed [86,112]. As pointed out above, such factors include physical activity, dietary intake, circadian rhythm, and musical expertise, which are rarely assessed in research on bilingualism and EF. At the same time, factors such as SES and cultural background, which have been shown to interact with bilingualism [38,89], play an important role in the development of EF. As Samuel and colleagues [37] point out, differences between East Asian and Western culture in educational practices and writing systems may be a confound in research on bilingualism and EF. Hence, as is already evident in recent research [73], a greater focus on individual differences may be necessary. Such individual differences could, for example, be described by assessing language usage patterns as indicated in the adaptive control hypothesis [43]. In this way, insight may be gained into within-group differences that are driven by distinct language interaction contexts [113].

(5) Underpowered research and statistical significance

It has been pointed out that mixed results in the literature of bilingual–monolingual differences might be partly due to Type 1 errors [15] since earlier studies documenting differences between bilinguals and monolinguals had used a rather small number of participants (for recent work with a larger number of participants, see Poarch [55]). While we agree that the power of a study needs to be sufficient, this does not necessarily mean large numbers of participants. Statistical power is related to effect size. Smaller effect sizes need more participants and trials, while larger effect sizes can make do with fewer. Also, Hope [114] notes that studies with larger numbers of participants are not necessarily always better given that power can sometimes be improved in smaller samples through ensuring more comparable groups. Furthermore, he adds, that better controlled, “smaller studies can be more informative than larger studies” [114] (p. 59). Therefore, power calculations run beforehand can assist in determining the ideal number of participants for a specific experiment. Furthermore, the still very prominent dependence on null-hypothesis significant testing (NHST) and its p-values to determine whether or not effects are significant may need to be reconsidered [115]. This may mean taking into account other statistical approaches such as Bayesian statistics [116,117] and focusing to a greater extent on effect sizes and confidence intervals, instead of solely the p-value and its arbitrary cut-off point of 0.05 to determine whether or not a difference is statistically significant and therefore important and meaningful. Again, while the above-mentioned is admittedly relevant for any research domain, given the subtle differences in EF task performance that are found in research comparing bilinguals and monolinguals, we believe this to be a relevant and pertinent issue.

(6) Cognitive real-life benefits of bilingualism

As mentioned above (see section “Cognitive advantages in real life”), small RT differences in EF tasks do not seem to be very relevant when considering life outside the research lab, particularly against the backdrop of the “bilingual advantage” discussion. However, structural and functional brain changes that bilingualism brings about can have an impact on real-life, exemplified in the aforementioned research on dementia onset and stroke recovery (see section “Neural differences”). Furthermore, enhanced skills such as perspective-taking in conversational settings [92], creative and divergent thinking [93], open-mindedness and cultural empathy [94], or tolerance of ambiguity [95] can have real effect on an individuals’ lives. These benefits might partly be due to differences in EF skills. A clearer effect of EF on real-life functioning can be found, for instance, in the literature on language processing, such as first language spelling skills [118] and language comprehension skills [119,120], where relationships with EF skills have been found. Studies such as these might be more informative with regards to real-life differences between bilingual and monolingual speakers and may assist in re-focusing the discussion on the “bilingual advantage” to a perspective that is more nuanced and one that takes into account effects on speakers’ daily lives.

## 9. Conclusions

In this paper, we asked whether or not it is advisable to maintain the notion of a bilingual advantage on non-verbal task performance, given the mixed results from research studies, the multitude of factors that have been found to affect cognitive functioning, and the possible lack of transfer of any cognitive differences found between groups to individuals’ real lives. After reviewing selected recent behavioural and neurophysiological research, we identified several relevant issues such as using longitudinal and within-group designs as well as re-evaluating the tasks used to tap cognitive processing in individuals. We believe that these recommendations should be considered in future research to move forward as a research community.

## References

[1] Kroll J.F., Bialystok E. (2013). Understanding the consequences of bilingualism for language processing and cognition. J. Cognit. Psychol..

[2] Bialystok E., Martin M.M., Viswanathan M. (2005). Bilingualism across the lifespan: The rise and fall of inhibitory control. Int. J. Biling..

[3] Kapa L.L., Colombo J. (2013). Attentional control in early and later bilingual children. Cognit. Dev..

[4] Poarch G.J., Van Hell J.G. (2012). Executive functions and inhibitory control in multilingual children: Evidence from second language learners, bilinguals, and trilinguals. J. Exp. Child Psychol..

[5] Poarch G.J., Bialystok E. (2015). Bilingualism as a model for multitasking. Dev. Rev..

[6] Blumenfeld H.K., Marian V. (2014). Cognitive control in bilinguals: Advantages in stimulus-stimulus inhibition. Biling. Lang. Cognit..

[7] Coderre E.L., van Heuven W.J.B., Conklin K. (2013). The timing and magnitude of Stroop interference and facilitation in monolinguals and bilinguals. Biling. Lang. Cognit..

[8] Colzato L.S., Bajo M.T., van den Wildenberg W.P.M., Paolieri D., Nieuwenhuis S.L.A., La Heij W., Hommel B. (2008). How does bilingualism improve executive control? A comparison of active and reactive inhibition mechanisms. J. Exp. Psychol. Learn. Mem. Cognit..

[9] Bialystok E., Poarch G.J., Luo L., Craik F.I.M. (2014). Effects of bilingualism and aging on executive function and working memory. Psychol. Aging.

[10] Adesope O.O., Lavin T., Thompson T., Ungerleider C. (2010). A systematic review and meta-analysis of the cognitive correlates of bilingualism. Rev. Educ. Res..

[11] Bialystok E. (2017). The bilingual adaptation: How minds accommodate experience. Psychol. Bull..

[12] Antón E., Duñabeitia J.A., Estévez A., Hernández J.A., Castillo A., Fuentes L.J., Davidson D.J., Carreiras M. (2014). Is there a bilingual advantage in the ANT task? Evidence from children. Front. Psychol..

[13] Duñabeitia J.A., Hernandez J.A., Antón E., Macizo P., Estevez A., Fuentes L.J., Carreiras M. (2014). The inhibitory advantage in bilingual children revisited: Myth or reality?. Exp. Psychol..

[14] Gathercole V.C.M., Thomas E.M., Kennedy I., Prys C., Young N., Guasch N.V., Roberts E.J., Hughes E.K., Jones L. (2014). Does language dominance affect cognitive performance in bilinguals? Lifespan evidence from preschoolers through older adults on card sorting, Simon, and rnetalinguistic tasks. Front. Psychol..

[15] Paap K.R., Greenberg Z.I. (2013). There is no coherent evidence for a bilingual advantage in executive processing. Cognit. Psychol..

[16] Darcy N.T. (1953). A review of the literature on the effects of bilingualism upon the measurement of intelligence. J. Genet. Psychol..

[17] Peal E., Lambert W.E. (1962). The relation of bilingualism to intelligence. Psychol. Monogr. Gener. Appl..

[18] Hakuta K. (1986). Mirror of Language: The Debate of Bilingualism.

[19] Bialystok E. (1988). Levels of bilingualism and levels of linguistic awareness. Dev. Psychol..

[20] Bialystok E., Harris R.J. (1992). Selective attention in cognitive processing: The bilingual edge. Cognitive Processing in Bilinguals.

[21] Galambos S.J., Hakuta K. (1988). Subject-specific and task-specific characteristics of metalinguistic awareness in bilingual children. Appl. Psycholinguist..

[22] Marian V., Spivey M. (2003). Competing activation in bilingual language processing: Within- and between-language competition. Biling. Lang. Cognit..

[23] Thierry G., Wu Y.J. (2007). Brain potentials reveal unconscious translation during foreign-language comprehension. Proc. Natl. Acad. Sci. USA.

[24] Keye D., Wilhelm O., Oberauer K., van Ravenzwaaij D. (2009). Individual differences in conflict-monitoring: Testing means and covariance hypothesis about the Simon and the Eriksen Flanker task. Psychol. Res..

[25] Botvinick M.M., Braver T.S., Barch D., Carter C.S., Cohen J.D. (2001). Conflict monitoring and cognitive control. Psychol. Rev..

[26] Diamond A. (2013). Executive functions. Annu. Rev. Psychol..

[27] Miyake A., Friedman N.P. (2012). The nature and organization of individual differences in executive functions: Four general conclusions. Curr. Direct. Psychol. Sci..

[28] Anderson P. (2002). Assessment and development of executive function (EF) during childhood. Child Neuropsychol..

[29] Mezzacappa E. (2004). Alerting, orienting, and executive attention: Developmental properties and sociodemographic correlates in an epidemiological sample of young, urban children. Child Dev..

[30] Noble K.G., McCandliss B.D., Farah M.J. (2007). Socioeconomic gradients predict individual differences in neurocognitive abilities. Dev. Sci..

[31] Best J.R. (2010). Effects of physical activity on children’s executive function: Contributions of experimental research on aerobic exercise. Dev. Rev..

[32] Hahn C., Cowell J.M., Wiprzycka U.J., Goldstein D., Ralph M., Hasher L., Zelazo P.D. (2012). Circadian rhythms in executive function during the transition to adolescence: The effect of synchrony between chronotype and time of day. Dev. Sci..

[33] Kuula L., Pesonen A.-K., Martikainen S., Kajantie E., Lahti J., Strandberg T., Tuovinen S., Heinonen K., Pyhälä R., Lahti M. (2015). Poor sleep and neurocognitive function in early adolescence. Sleep Med..

[34] Kim J.Y., Wang S.W. (2017). Relationships between dietary intake and cognitive function in healthy Korean children and adolescents. J. Lifestyle Med..

[35] Peretz I., Zatorre R.J. (2005). Brain organization for music processing. Annu. Rev. Psychol..

[36] Zuk J., Benjamin C., Kenyon A., Gaab N. (2014). Behavioral and neural correlates of executive functioning in musicians and non-musicians. PLoS ONE.

[37] Samuel S., Roehr-Brackin K., Pak H., Kim H. (2018). Cultural effects rather than a bilingual advantage in cognition: A review and an empirical study. Cognit. Sci..

[38] Tran C.D., Arredondo M.M., Yoshida H. (2015). Differential effects of bilingualism and culture on early attention: A longitudinal study in the U.S., Argentina, and Vietnam. Front. Psychol..

[39] Bak T., Robertson I. (2016). Biology enters the scene—A new perspective on bilingualism, cognition, and dementia. Neurobiol. Aging.

[40] Bak T. (2016). Cooking pasta in La Paz. Linguist. Approaches Biling..

[41] Filippi R., Morris J., Richardson F.M., Bright P., Thomas M.S.C., Karmiloff-Smith A., Marian V. (2015). Bilingual children show an advantage in controlling verbal interference during spoken language comprehension. Biling. Lang. Cognit..

[42] Anderson J.A.E., Chung-Fat-Yim A., Bellana B., Luk G., Bialystok E. (2018). Language and cognitive control networks in bilinguals and monolinguals. Neuropsychologica.

[43] Green D.W., Abutalebi J. (2013). Language control in bilinguals: The adaptive control hypothesis. J. Cognit. Psychol..

[44] Calabria M., Costa A., Green D.W., Abutalebi J. (2018). Neural basis of bilingual language control. Ann. N. Y. Acad. Sci..

[45] Antoniou M. (2019). The advantages of bilingualism. Annu. Rev. Linguist..

[46] Eriksen B.A., Eriksen C.W. (1974). Effects of noise letters upon the identification of a target letter in a nonsearch task. Percept. Psychophys..

[47] Simon J.R., Rudell A.P. (1967). Auditory S–R compatibility: The effect of an irrelevant cue on information processing. J. Appl. Psychol..

[48] Prior A., MacWhinney B. (2010). A bilingual advantage in task switching. Biling. Lang. Cognit..

[49] Friedman N.P., Miyake A. (2004). The relations among inhibition and interference control functions: A latent-variable analysis. J. Exp. Psychol. Gener..

[50] Fan J., McCandliss B.D., Sommer T., Raz A., Posner M.I. (2002). Testing the efficiency and independence of attentional networks. J. Cognit. Neurosci..

[51] Park J., Ellis Weismer S., Kaushanskaya M. (2018). Changes in executive function over time in bilingual and monolingual school-aged children. Dev. Psychol..

[52] Zelazo P.D. (2006). The Dimensional Change Card Sort (DCCS): A method of assessing executive function in children. Nat. Protocols.

[53] Bialystok E., Martin M.M. (2004). Attention and inhibition in bilingual children: Evidence from the dimensional change card sort task. Dev. Sci..

[54] Carlson S.M., Meltzoff A.N. (2008). Bilingual experience and executive functioning in young children. Dev. Sci..

[55] Poarch G.J. (2018). Multilingual language control and executive function: A replication study. Front. Commun..

[56] Naeem K., Filippi R., Periche-Tomas E., Papageorgiou A., Bright P. (2018). The importance of socioeconomic status as a modulator of the bilingual advantage in cognitive ability. Front. Psychol..

[57] Christoffels I.K., de Haan A.M., Steenbergen L., van den Wildenberg W.P.M., Colzato L.S. (2015). Two is better than one: Bilingual education promotes the flexible mind. Psychol. Res..

[58] Garbin G., Sanjuan A., Forn C., Bustamante J.C., Rodriguez-Pujadas A., Belloch V., Hernandez M., Costa A., Avila C. (2010). Bridging language and attention: Brain basis of the impact of bilingualism on cognitive control. NeuroImage.

[59] Prior A., Gollan T.H. (2011). Good Language-Switchers are Good Task-Switchers: Evidence from Spanish-English and Mandarin-English Bilinguals. J. Int. Neuropsychol. Soc..

[60] Stasenko A., Matt G.E., Gollan T.H. (2017). A relative bilingual advantage in switching wiith preparation: Nuanced explorations of the proposed association between bilingualism and task switching. J. Exp. Psychol. Gener..

[61] Paap K.R., Myuz H.A., Anders R.T., Bockelman M.F., Mikulinsky R., Sawi O.M. (2017). No compelling evidence for a bilingual advantage in switching or that frequent language switching reduces switch cost. J. Cognit. Psychol..

[62] Lehtonen M., Soveri A., Laine A., Jarvenpaa J., de Bruin A., Antfolk J. (2018). Is bilingualism associated with enhanced executive functioning in adults? A meta-analytic review. Psychol. Bull..

[63] Bialystok E., Craik F.I.M., Luk G. (2012). Bilingualism: Consequences for mind and brain. Trends Cognit. Sci..

[64] Valian V. (2015). Bilingualism and cognition. Biling. Lang. Cognit..

[65] Alladi S., Bak T.H., Duggirala V., Surampudi B., Shailaja M., Shukla A.K., Chaudhuri J.R., Kaul S. (2013). Bilingualism delays age at onset of dementia, independent of education and immigration status. Neurology.

[66] Bialystok E., Craik F.I.M., Freedman M. (2007). Bilingualism as a protection against the onset of symptoms of dementia. Neuropsychologia.

[67] Alladi S., Bak T.H., Mekala S., Rajan A., Chaudhuri J.R., Mioshi E., Krovvidi R., Surampudi B., Duggirala V., Kaul S. (2016). Impact of bilingualism on cognitive outcome after stroke. Stroke.

[68] Paplikar A., Mekala S., Bak T.H., Dharamkar S., Alladi S., Kaul S. (2019). Bilingualism and the severity of poststroke aphasia. Aphasiology.

[69] Del Maschio N., Fedeli D., Abutalebi J. (2018). Bilingualism and aging: Why research should continue. Linguist. Approaches Biling..

[70] Maguire E.A., Gadian D.G., Johnsrude I.S., Good C.D., Ashburner J., Frackowiak R.S.J., Frith C.D. (2000). Navigation-related structural change in the hippocampi of taxi drivers. Proc. Natl. Acad. Sci. USA.

[71] Bermudez P., Lerch J.P., Evans A.C., Zatorre R.J. (2008). Neuroanatomical correlates of musicianship as revealed by cortical thickness and voxel-based morphometry. Cereb. Cortex.

[72] Pliatsikas C. (2019). Understanding structural plasticity in the bilingual brain: The Dynamic Restructuring Model. Biling. Lang. Cogn..

[73] DeLuca V., Rothman J., Bialystok E., Pliatsikas C. (2019). Redefining bilingualism as a spectrum of experiences that differentially affects brain structure and function. Proc. Natl. Acad. Sci. USA.

[74] Grundy J.G., Anderson J.A., Bialystok E. (2017). Neural correlates of cognitive processing in monolinguals and bilinguals. Ann. N. Y. Acad. Sci..

[75] Olsen R.K., Pangelinan M.M., Bogulski C., Chakravarty M.M., Luk G., Grady C.L., Bialystok E. (2015). The effect of lifelong bilingualism on regional grey and white matter volume. Brain Res..

[76] Gold B.T., Kim C., Johnson N.F., Kryscio R.J., Smith C.D. (2013). Lifelong bilingualism maintains neural efficiency for cognitive control in aging. J. Neurosci..

[77] Coderre E.L., van Heuven W.J.B. (2014). Electrophysiological explorations of the bilingual advantage: Evidence from a Stroop task. PLoS ONE.

[78] Kousaie S., Phillips N.A. (2012). Conflict monitoring and resolution: Are two languages better than one? Evidence from reaction time and event-related brain potentials. Brain Res..

[79] van den Noort M., Struys E., Bosch P., Jaswetz L., Perriard B., Yeo S., Barisch P., Vermeire K., Lee S.-H., Lim S. (2019). Does the bilingual advantage in cognitive control exist and if so, what are its modulating factors? A systematic review. Behav. Sci..

[80] Blanco-Elorrieta E., Pylkänen L. (2018). Ecological validity in bilingualism research and the bilingual advantage. Trends Cognit. Sci..

[81] Blom E., Boerma T., Bosma E., Cornips L., Everaert E. (2017). Cognitive advantages of bilingual children in different sociolinguistic contexts. Front. Psychol..

[82] Crivello C., Kuzyk O., Rodrigues M., Friend M., Zesiger P., Poulin-Dubois D. (2016). The effects of bilingual growth on toddlers’ executive function. J. Exp. Child Psychol..

[83] De Cat C., Gusnanto A., Serratrice L. (2018). Identifying a threshold for the executive function advantage in bilingual children. Stud. Second Lang. Acquis..

[84] Ladas A.I., Carroll D.J., Vivas A.B. (2015). Attentional processes in low-socioeconomic status bilingual children: Are they modulated by the amount of bilingual experience?. Child Dev..

[85] Thomas-Sunesson D., Hakuta K., Bialystok E. (2018). Degree of bilingualism modifies executive control in Hispanic children in the US. Int. J. Biling. Educ. Biling..

[86] Baum S., Titone D. (2014). Moving toward a neuroplasticity view of bilingualism, executive control, and aging. Appl. Psycholinguist..

[87] Poarch G.J., Van Hell J.G., Wiebe S., Karbach J. (2017). Bilingualism and the development of executive function in children: The interplay of languages and cognition. Executive Function: Development Across the Life Span (Frontiers of Developmental Science).

[88] Kuppens A.H. (2010). Incidental foreign language acquisition from media exposure. Learning. Media Technol..

[89] Engel de Abreu P.M., Cruz-Santos A., Tourinho C.J., Martin R., Bialystok E. (2012). Bilingualism enriches the poor: Enhanced cognitive control in low-income minority children. Psychol. Sci..

[90] Kempert S., Saalbach H., Hardy I. (2011). Cognitive benefits and costs of bilingualism in elementary school students: The case of mathematical word problems. J. Educ. Psychol..

[91] Poarch G.J. (2017). Bialystok Assessing the implications of migrant multilingualism for language education. Z. Erzieh..

[92] Schroeder S.R. (2018). Do bilinguals have an advantage in Theory of Mind? A meta-analysis. Front. Commun..

[93] Kharkhurin A.V. (2009). The role of bilingualism in creative performance on divergent thinking and invented alien creatures tests. J. Creat. Behav..

[94] Dewaele J.M., Stavans A. (2014). The effect of immigration, acculturation and multicompetence on personality profiles of Israeli multilinguals. Int. J. Biling..

[95] Dewaele J.M., Wei L. (2013). Is multilingualism linked to a higher tolerance of ambiguity?. Biling. Lang. Cognit..

[96] Goetz P.J. (2003). The effects of bilingualism on theory of mind development. Biling. Lang. Cognit..

[97] Kovács Á.M. (2009). Early bilingualism enhances mechanisms of false-belief reasoning. Dev. Sci..

[98] Baron-Cohen S., Leslie A.M., Frith U. (1985). Does the autistic child have a “theory of mind”?. Cognition.

[99] Diaz V., Farrar M.J. (2018). Do bilingual and monolingual preschoolers acquire false belief understanding similarly? The role of executive functioning and language. First Lang..

[100] Fan S.P., Liberman Z., Keysar B., Kinzler K.D. (2015). The exposure advantage: Early exposure to a multilingual environment promotes effective communication. Psychol. Sci..

[101] Wilson R.S., Beckett L.A., Barnes L.L., Schneider J.A., Bach J., Evans D.A., Bennett D.A. (2002). Individual differences in rates of change in cognitive abilities of older persons. Psychol. Aging.

[102] Poarch G.J., Van Hell J.G., Sekerina I., Valina V., Spradlin L. (2019). Does performance on executive function tasks correlate? Evidence from child trilinguals, bilinguals, and second language learners. Bilingualism, Executive Function, and Beyond: Questions and Insights [Studies in Bilingualism 57].

[103] Mansfield K.L., van der Molen M.W., Falkenstein M., van Boxtel G.J. (2013). Temporal dynamics of interference in Simon and Eriksen tasks considered within the context of a dual-process model. Brain Cognit..

[104] Costa A., Hernández M., Costa-Faidella J., Sebastián-Gallés N. (2009). On the bilingual advantage in conflict processing: Now you see it, now you don’t. Cognition.

[105] Qu L., Low J.J.W., Zhang T., Li H., Zelazo P.D. (2016). Bilingual advantage in executive control when task demands are considered. Biling. Lang. Cognit..

[106] Colsher P., Wallace R. (1991). Longitudinal application of cognitive function measures in a defined population of community-dwelling elders. Ann. Epidemiol..

[107] Rueda M.R., Fan J., McCandliss B.D., Halparin J.D., Gruber D.B., Lercari L.P., Posner M.I. (2004). Development of attentional networks in children. Neuropyschologica.

[108] Zhou B., Krott A. (2016). Data trimming procedure can eliminate bilingual cognitive advantage. Psychon. Bull. Rev..

[109] Zhou B., Krott A. (2018). Bilingualism enhances attentional control in non-verbal conflict tasks—Evidence from ex-Gaussian analyses. Biling. Lang. Cognit..

[110] Poarch G.J., Vanhove J., Berthele R. (2019). The effect of bidialectalism on executive function. Int. J. Biling..

[111] Open Science Collaboration (2015). Estimating the reproducibility of psychological science. Science.

[112] Van Hell J.G., Poarch G.J. (2014). How much bilingual experience is needed to affect executive control?. Appl. Psycholinguist..

[113] Yang H., Hartanto A., Yang S. (2016). The complex nature of bilinguals’ language usage modulates task-switching outcomes. Front. Psychol..

[114] Hope T.M.H. (2015). The bilingual cognitive advantage: No smoke without fire?. AIMS Neurosci..

[115] McShane B.B., Gal D., Gelman A., Robert C., Tackett J.L. (2017). Abandon statistical significance. arXiv.

[116] Dienes Z. (2016). How Bayes factor change scientific practice. J. Math. Psychol..

[117] Wagenmakers E.M., Morey R.D., Lee M.D. (2016). Bayesian benefits for the pragmatic researcher. Curr. Direct. Psychol. Sci..

[118] Czapka S., Klassert A., Festman J. (2019). Executive functions and language: Their differential influence on mono- vs. multilingual spelling in primary school. Front. Psychol..

[119] Litcofsky K.A., Tanner D., van Hell J.G. (2015). Effects of language experience, use, and cognitive functioning on bilingual word production and comprehension. Int. J. Biling..

[120] Navarro-Torres C., Lopez Garcia D., Chidambaram V., Kroll J.F. (2019). Cognitive Control Facilitates Attentional Disengagement during Second Language Comprehension. Brain Sci..

